# The Impact of COVID-19 on Māori Patients Attending Diabetes Annual Review

**DOI:** 10.7759/cureus.63571

**Published:** 2024-07-01

**Authors:** Natalie Burkhardt, Claudia Gomez Fernandez, Donna Dunn

**Affiliations:** 1 Family and Community Medicine, Oceania University of Medicine, Whangarei, NZL; 2 Family Medicine, Devonport Naval Base, Auckland, NZL; 3 General Practice, Devonport Naval Base Medical Center, Auckland, NZL

**Keywords:** covid-19, general practitioner, distrust, diabetes mellitus, indigenous maori, colonization

## Abstract

Māori, the indigenous population of New Zealand, represent 17.1% of the country’s population (Statistics New Zealand 2021) and are over-represented in all negative indices. In particular, Māori are underprivileged in terms of socioeconomics and health due to the residual effects of colonization. The global COVID-19 pandemic, caused by the novel coronavirus SARS-CoV-2, has been one of the most significant public health crises in modern history. Vulnerable populations, such as the elderly and those with underlying health conditions, were and remain at higher risk of severe outcomes. In the New Zealand context and given the health statistics, Māori were identified as a group that was at high risk from COVID-19. Using a mixed method approach, we attempt to identify the reasons why a cohort of New Zealand Māori with type II diabetes mellitus (DM II) and a history of regular attendance failed their Diabetes Annual Review (DAR) post-COVID-19.

Twelve Māori participants were recruited (> 18 years) from a Māori Diabetes database of an urban General Practitioners (GP) Clinic in Northland. A 9-point questionnaire and an unstructured telephone conversation utilizing a Kaupapa Māori (Māori philosophy) approach were utilized, and data were collated.

Findings suggest the New Zealand government’s COVID-19 vaccine mandates served to exacerbate Māori distrust of health professionals. Trust is the foundation of every successful relationship whether it be business, professional, health, or personal. Health delivery and uptake are based on this foundation. Whatever the reason for the loss of trust in the medical profession, historical colonial trauma, swayed by conspiracy theory, or otherwise, considering this factor should influence the structure and approach of public health initiatives directed toward Indigenous people internationally.

## Introduction

Based on historical precedence, economic downturns disproportionately impact disadvantaged populations in Western societies, especially indigenous peoples with colonial histories [1,2]. Identifying emergent barriers among this patient population may provide valuable insights and understandings to inform the development of current and future health strategies at both community and governmental levels. The repercussions of the COVID-19 pandemic on indigenous populations have the potential to exacerbate the negative effects wrought by colonization [3].

It was expected that the COVID-19 pandemic would have far-reaching and negative implications, especially for groups such as Māori that were identified as vulnerable and at severe risk from the virus. Representing 17.1% of the total New Zealand population [4], Māori feature heavily in all negative indices. In terms of disparities, Māori are underprivileged in socioeconomics and health due to the residual effects of colonization, which is borne out in the literature [5-7].

Using a mixed method approach, this study aims to understand why a group of Māori individuals with type II diabetes mellitus (DM II), who prior to the COVID-19 pandemic, had a regular attendance history, failed their Diabetes Annual Review (DAR) from May 2021 to early August 2022.

## Materials and methods

Within the context of the excess burden of DM II and Māori resistance to the health system, it was hypothesized that COVID-19 would adversely affect Māori patients' access to DARs during the study period (May 2021 to August 2022). This study utilized Kaupapa Māori Research - by Māori, with Māori, and for Māori - that seeks to benefit Māori by critiquing socio-political systems that create ethnic inequities by rejecting victim blaming and deficit [8]. Kaupapa Māori is derived from Māori epistemologies involving complex relationships with the physical and metaphysical world. This influences how they interact with the world and organize their society [9]. About research involving Māori participants, this concept of Kaupapa Māori provides a platform for structuring research methodology to gain a more succinct approach and a more accurate way of interpreting the data gained.

After approaching three Family Medicine/General Practitioner Practices within a 10km radius, the study was finally conducted in collaboration with one clinic where the practice manager granted permission to access their Māori diabetes database. Twelve Māori participants (>18 years) were recruited from a Māori Diabetes database at an urban clinic in Northland. Selection was based on the following criteria: diagnosis of DM II for more than two years, participation in pre-COVID-19 for at least two years, and ability to be contacted by telephone. Primary data were collected using a mixed-method approach using a 9-point questionnaire (appendix 2) followed by 30-45-minute unstructured interviews using the Kaupapa Māori approach) to embrace the broader social, cultural, spiritual, and familial aspects influencing perceived barriers to accessing/attending their DARs.

Initial contact with the participants was via an introduction phone call. After explaining the reason for the phone call, and the objective and method of the study, verbal consents were obtained from the 12 participants and a follow-up phone call was scheduled at their convenience for data collection. The DAR, which is free of charge to patients with DM II, regardless of ethnicity is a critical component of diabetes management in New Zealand family medicine and facilitates the assessment of glycemic control and earlier detection and intervention of diabetes-related complications. Hence, engaging and monitoring DM II patients to ensure consistent attendance to their recalls is essential for disease management. Identifying reasons related to COVID-19's impact on Māori patients attending their DARs may shed light on valuable data influencing patient engagement.

The post-questionnaire interviews served to provide qualitative insight into how the COVID-19 pandemic affected their access to and engagement with their diabetic health providers. This mixed-method approach ensured a comprehensive understanding of the research questions from both quantitative and qualitative perspectives and to provide a holistic approach.

Ethical approval for this study was granted by the Oceania University of Medicine Ethics Committee (22-0315NB). Ensuring ethical rigor and cultural sensitivity was paramount throughout the research process. The principles of Kaupapa Māori Research were adhered to at all stages, ensuring that the research was conducted in a manner that respects and upholds values and traditions in Māori culture.

## Results

The results extracted from the data are taken from the “Agreed” results. The other “neutral” and “disagree” answers were not presented as a percentage, only the Agreed. In the agreed results, lack of trust, whanau commitments taking priority over your own health, perceived change in doctor/patient relationship during COVID-19, and patients not willing to see another doctor besides their own were at 66.7%. The indicator in the most influential of these four reasons, in particular, was the contradicting disagree at 16.7% for lack of trust and 33.3% for lack of trust, putting more weight on lack of trust.

Interestingly, finance, which has traditionally been a strong indicator influencing Māori access to healthcare, was not significant with only 8.3% agreeing. The reason for this may be borne from the financial support provided by the government during COVID-19 with more than one participant sharing that they had been in a better financial position during the pandemic due to government support. Due to the small cohort, this should be interpreted with caution.

**Figure 1:**
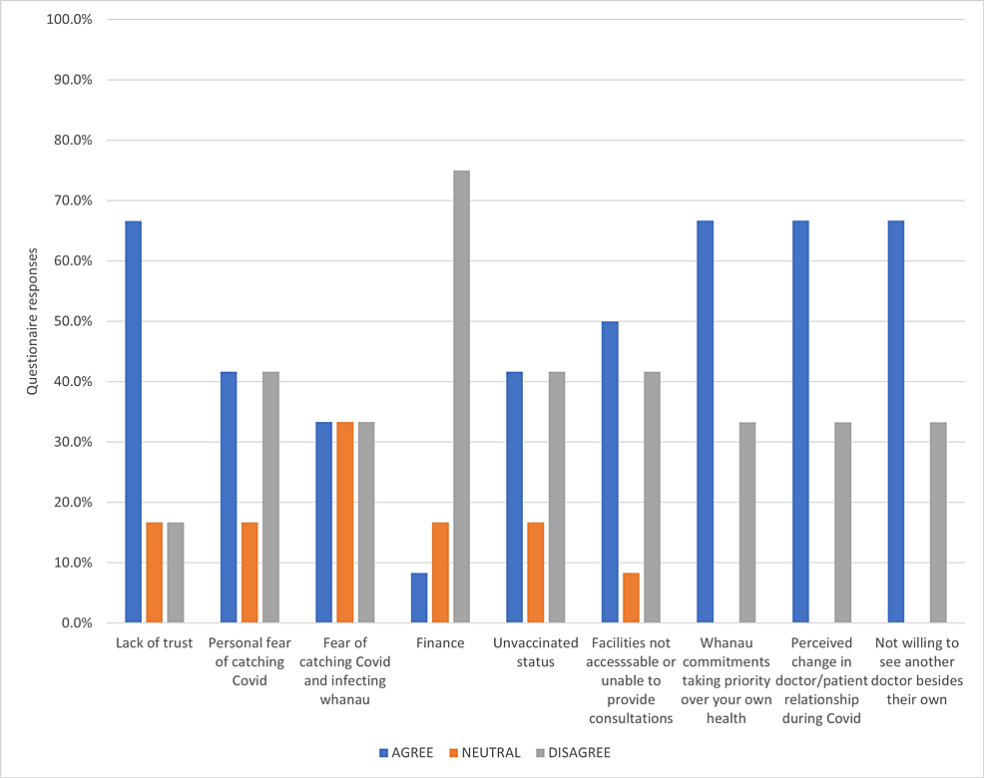
Reasons for failing to attend Diabetes Annual Reviews during the study period (COVID-19 pandemic, May 2021 to August 2022).

As outlined in Figure 1, the other reasons for failing DARs were not as influential as the above-mentioned. Seven of the 12 study participants declared they had received the COVID-19 vaccines, and although not explicitly included in the questionnaire, vaccination mandates proved a highly emotive issue that affected participants' engagement in, and perspective of trust in the healthcare system. The five unvaccinated participants expressed trepidation over ongoing pressure from healthcare workers to comply with the vaccine mandates while several vaccinated participants described their resentment about having been pressured into getting vaccinated.

Lack of trust (66.7%)

The majority of participants made comments about their loss of trust in and lack of sincerity in healthcare workers. Comments, such as the following, were common.

“They just want to tick their boxes”.

“They don’t do anything; just give you pills.”

“They just want us to come back on checks so they can charge us.”

“I haven’t had the ‘jab’ yet and they’ll push me into having it.”

Personal fear of contracting COVID-19 (41.6%)

This issue received very little attention during post-questionnaire discussions.

Fear of catching COVID-19 and spreading it to whānau at home (33.3%)

While a few participants expressed concerns about infecting whānau, many participants regardless of their vaccination status existed within non-vaccinated whānau environments.

Finance (8.3%)

This result was unexpected and potentially reflects the availability of government COVID-19-related assistance:

“We’re better off now than before COVID-19 not having to work and we’re getting paid.”

Unvaccinated status (41.6%)

While some unvaccinated participants associated their unvaccinated status with a sense of autonomy, others feared being pressured by healthcare providers to improve low regional Māori vaccination rates:

“If I go to the doctors, they just won’t leave the vaccine thing alone.”

“I’m not having it (the vaccine) that's it. I’m in control of what’s happening to me.”

Facility inaccessible (50%)

Among the participants who contacted the clinic via teleconsultation with health providers during COVID-19, negative perceptions regarding the absence of “kanohi ki te kanohi” or face-to-face interaction were found. Face-to-face communication is culturally significant for Māori as there is a concept that it allows an accurate interpretation of the communication allowing the person not only to see, but also to hear and feel the relationship [10].

Whānau commitments taking priority over personal healthcare (66.7%)

Although the inability to leave elderly relatives at home was frequently expressed, COVID-19 infections within whānau were not reported. The need to help whānau look after children was also common. 

Perceived change in doctor-patient relations during COVID-19 (66.7%)

Similarly, there was a convergence of reporting by vaccinated and unvaccinated participants that pressure around vaccinations had adversely affected their doctor-patient relations.

Not willing to see another doctor (66.7%)

While 66.7% of participants were unwilling to see another GP, the remainder seemed ambivalent regarding who they interacted with - whether it was a nurse or any other GP. These participants, however, expressed frustration with the quality of care from others, particularly the lack of accumulated knowledge about them.

“I ended up speaking to a nurse or someone else who didn’t know anything about me.”

“You never get the same one anyway, and the new one never knows what your pills are.”

“You never get the doctor; you just talk to the nurse.”

“It’s not like being in the same room, yeah it’s hard to talk about stuff on the phone.”

“It’s just money-making, you get charged for talking to someone.”

Participants needed reassurance that all interactions would be confidential and that they would remain anonymous to staff at the clinic: “You won’t tell them this stuff (the doctor) will you?”

Thereafter, all participants were very willing to explain their reasons for not attending DARs and share their experiences and perceptions of the current health system and ‘modern medicine.’ Of concern, a strong message of apathy was communicated by many participants.

## Discussion

Historically, distrust from Māori regarding the Western Health System has been identified and well-documented as a barrier to engagement. Our study findings identified this as a major factor that influenced Māori access to primary healthcare around COVID-19 and this cohort's DARs. Other expected barriers such as financial, access, and whanau responsibility among other reasons did not seem to influence their DAR.

Successive governments have made legislative attempts to close the gaps in ethnic health outcomes, but have failed to meet their objectives [11,12]. Poor service uptake by Māori has been attributed to a perceived lack of provider respect, repeated mispronunciation of Māori names; distrust; comprehension difficulties; and feelings they and their problems were not significant [13-16]. The Waitangi Tribunal Claim Wai 2575 (Health Services and Outcomes Inquiry Kaupapa) [17] comprehensively set out the “Māori experience” of the health system. In 2020, over 220 claims were received seeking participation in the Health Services and Outcomes Inquiry Kaupapa. Claim Wai 2575 covered both historical and contemporary times, a range of issues relating to the health system, specific health services and outcomes as well as institutional racism and unfair treatment inside the health system. Claim Wai 2575 was also the motivator to the Pae Ora (Healthy Futures) Act 2022 that centers on recognizing the Tiriti o Waitangi (Treaty of Waitangi), priority populations (traditionally marginalized groups such as Māori, Pacifica, disabled, and LGBTQI) and equity. As of the time of writing this article, the current health reforms are still underway. As outlined in Claim Wai 2575, the “Māori experience” is one of the inequities in physical and mental illness health statistics [8,12,18], excess rates of Māori mortality and morbidity from chronic diseases compared to non-Māori [19], historical trauma associated with the intergenerational loss of tribal lands and the suppression of Māori culture and language [12,20].

About the context of our study, the historical reasons implicated for compromised uptake of medical services by Māori are still influencers but the lack of trust in the health system appears to have surpassed risk factors such as socioeconomic disadvantage, and poor educational outcomes, previously used as markers of inequitable health outcomes for Māori.

New Zealand took early action by using its remote geographical location and isolation to implement strict border controls and travel restrictions early into the pandemic, including mandatory quarantine for all incoming travelers. This helped limit the introduction of the virus into the country [21]. In March 2020, New Zealand implemented one of the strictest lockdowns in the world, including the closure of non-essential businesses and schools, and restrictions on movement. It was the first time since World War II that New Zealand closed its borders [22]. This approach involved strict border controls, testing, and contact tracing to identify and isolate cases [23]. The Labour government provided financial support to individuals and businesses affected by the pandemic, including wage subsidies and assistance for essential workers. Efforts were made to ensure that vulnerable populations, such as the elderly and Māori communities, had access to healthcare and support services. New Zealand rolled out a nationwide vaccination campaign, prioritizing high-risk populations and frontline workers [24].

It was expected COVID-19 would exacerbate the converging risks of increased whānau obligations, rising unemployment, and resultant resource sharing. Despite increasing rates of Māori unemployment in early COVID-19 statistics [25], study participants did not report socioeconomic disadvantage as the reason for DARs non-attendance. While this result was unexpected, it potentially reflects participants’ access to government COVID-19 relief funding for Māori and other disadvantaged groups. A qualitative study by Choi et al. of low-income individuals (n = 42) interviewed in June-July 2020 immediately after the lockdown reported coping financially while still experiencing financial stress [26].

New Zealand’s welfare state ensured participants had access to health services and welfare payments, but there were challenges. Welfare payments did not, however, fully meet participants’ needs, and support from charitable organizations was critical for some whanau. While participants acknowledged the raised payments across all government benefits and the increased Winter Energy Payment, a disproportionate number of Māori participants stated that they endured financial stress, which in some cases influenced access to medical services [26]. Although this study identified very little financial impact, one participant reported having to choose between paying for their medical scripts or paying the bills.

Globally, the low socioeconomic demographic has borne a disproportionate burden of the pandemic and State responses to COVID-19 appear to have exacerbated disparities between indigenous and non-indigenous health outcomes [2,27]. Although participants expressed a strong desire for continuity of the doctor-patient relations, satisfaction appeared conditioned by a desire for individualized treatment and the absence of a paternal approach to their management of DM II [28]. A qualitative study by Romana et al. [29] found although the New Zealand pandemic approach effectively mobilized the nation into a swift lockdown, significantly reducing transmission, while applauded internationally, served to further marginalized communities already under stress.

Māori are disproportionately burdened by DM II with earlier onset and higher rates of prevalence, comorbidities, morbidity, and mortality than non-Māori [30]. Given the above, including both the historical and contemporary position of Māori, it was reasonable to expect that the COVID-19 pandemic would negatively affect Māori access to DARs. The New Zealand Government built its COVID-19 strategy, in part, on the threat to vulnerable populations such as Māori. As outlined in Claim Wai 2575, there are critical treatment gaps across the continuum of risk and disease persist, including the assessment and management of diabetes risk in Māori and time-critical access to and receipt of acute and future follow-up services. Health clinics engaging Māori patients with DM II and their whānau throughout the COVID-19 pandemic was crucial to the maintenance and prevention of diabetes-associated complications [31]. From this perspective, maintaining ties between Māori patients, primary healthcare professionals, and the health system is crucial [32,33]. While the New Zealand Government’s “rapid response” policy to the COVID-19 pandemic was praised internationally for its low mortality rate, containment of the outbreak through border closures and lockdown as well as a mandated roll-out of the vaccine, the national backlash to these measures resulted in civil unrest and a change of government. The COVID-19 pandemic evoked powerful personal responses at home and abroad as individuals reflected on their mortality, values, and priorities within their cultural frameworks [34].

For Māori, a population already vulnerable, evidenced by over-representation in all of the negative indices and combined with an already established mistrust of the health system, findings from this small qualitative study suggest the New Zealand government’s rapid and effective pandemic response further exacerbated distrust among participants. As a result, former adherence to diabetes guidelines to reinforce not only maintaining their health and bodily autonomy were put at risk. In the future, it is urged that Government agencies consider these factors when chartering any public health actions and policies because of the real risk of jeopardizing the health gains Māori have made in managing their health.

While community engagement and trust in government institutions played a crucial role in the New Zealand government’s strategy for the COVID-19 pandemic, the Māori experience, especially of the health system, is somewhat different. The delivery of Western healthcare has long been challenged by Māori whānau (extended family living arrangements), customs, and culture [28].

Despite the colonial imposition of the Western concept of the nuclear family, Māori continue to live in whānau or extended family relationships, informed by Kaupapa Māori a “body of knowledge accumulated by the experiences through the history of the Māori people,” underpinning the way Māori people think, understand, interact and interpret the world [9,35].

Historically, a lack of resources or past difficulties engaging with Māori were identified as barriers to improving inequities in Māori health [18]. Critical, however, treatment gaps across the continuum of risk and disease persist, including the assessment and management of diabetes risk in Māori and time-critical access to and receipt of acute and future follow-up services. Health clinic's engagement of Māori patients with DM II and their whānau throughout the COVID-19 pandemic was crucial to the maintenance and prevention of diabetes-associated complications [31]. The prognosis for patients contracting COVID-19 was poorer if the pre-existing disease was present: respiratory, cardiovascular, diabetes, autoimmune disorders, etc. Given the higher burden of these types of diseases for Māori, which is well-covered in the literature, managing these conditions was essential in avoiding the sequelae of complications, such as respiratory failure, cardiovascular failure, and cerebrovascular incidents (stroke, seizures, and bleeds) in patients with COVID-19.

As our study was based on a particular sector of health (family health) rather than other health streams like surgery, psychiatry, and so forth, our study found Māori distrust of GPs. Whether related to GPs personally or what they represented, together with a palpable disdain for the perceived paternalistic approach to their health rather than doctor-patient partnership was the major influencer for the lack of recall attendance.

Trust is the foundation of every successful relationship whether it be business, professional, health, or personal. Health delivery and uptake are based on this foundation. Whatever the reason for the continued lack of trust in the medical profession: historical colonial trauma, swayed by conspiracy theory, or otherwise, considering this factor needs to be at the core of health policy and influence the structure and approach of public health initiatives directed toward Indigenous people internationally.

Trust and vaccine mandate

Although the questionnaire prompted the issue of trust, insights that emerged during interviews revealed a strong distrust of and apathy towards the wider medical system including health professionals rather than the COVID-19 pandemic per se. This was not the expected result. Participants guided post-questionnaire conversations and expressed discontent with the vaccine mandate. Almost half of the participants (5/12) reported that although they did not want to be vaccinated, they felt pressured into doing so; a trend identified among other indigenous groups [36].

Reporting from District Health Boards identified several regions marked by high area derivation and high-density Māori populations, including Northland, as areas experiencing slow COVID-19 vaccine uptakes. While low vaccination rates among Māori were potentially underpinned by a subconscious distrust of the Western health system and concomitant loss of personal and cultural autonomy [37], the effect of conspiracy theories regarding the efficacy of the COVID-19 vaccine can only be speculated at. Study participants’ fears of contracting COVID-19 and infecting immediate and extended family members were well-founded, given excess regional rates of Māori house crowding and the high percentage of elderly Māori with comorbidities living with whānau. Research identifying that Māori have a substantially higher risk of hospitalization for COVID-19 and prior estimates that Māori would experience higher infection fatality rates from COVID-19 contributed towards the prioritization of Māori as a group for vaccination against COVID-19 [2].

Vaccine uptake

Research on Māori vaccine uptake is mixed [38] and found the vaccination uptake gap among Māori and Pacific people, compared to the general population was, slow. A prospective study by Prickett, et al, [39], found that while a majority (71%) intended to take the COVID-19 vaccine once available, a sizeable minority of participants were identified as young, female, and less educated, were unsure (15%) about or unlikely (14%) to get the vaccine, primarily because of perceptions of unknown future side effects. Ethnicity was not significantly associated with COVID-19 vaccine hesitancy [39] which underscores the need to understand the shifting public vaccine intentions to increase vaccination uptake [36]. Internationally, New Zealand’s implementation of a rapid and effective lockdown, high rates of vaccinations, and low rates of COVID-19-related mortality were considered successful [40]. Nonetheless, Whitehead et al. [41] found spatial access to vaccination services disadvantaged high-priority populations including Māori, Pasifika people, over 65-year-olds, and rural residents. In their review of localized Māori community responses to COVID-19 lockdowns, Cassim and Keelan found that while the lockdowns challenged the health of whānau Māori (Māori families), alongside their social, cultural, and financial well-being, Māori repeatedly demonstrated innovative resilience throughout the pandemic [42]. It is important to note, however, that the majority of Māori are vaccinated (approximately 70%), although there is variation between regions. The vaccination roll-out was unequal in that remote geographical regions did not have the same access as those in urban centers. Also, Iwi-Māori groups were at the forefront of the COVID-19 pandemic, providing assistance to those individuals and their whānau affected by illness [43]. From this data, it would be difficult to imply that the pandemic enhanced the historical lack of trust in the health system.

Moving forward, future health policy and health initiatives with the objective of addressing the health disparities between Māori and non-Māori may need to be focused on regaining and establishing a sound foundation of trust and acceptance in the current health system rather than providing all the health resources at great public expense. How this is incorporated into health policy is difficult to foresee but continuing to increase the number of Māori health professionals who have cultural insight and can approach Māori health in a “Kaupapa Māori” light could have a significant impact. In parallel, inviting respected cultural leaders to partner in local policy development may provide a segway to addressing the low uptake of health services by Māori.

Limitations

This study had several limitations. First, data were accessed from the database of a single GP. It would have been interesting to a) compare results from multiple GPs; b) use data from several Māori and non-Māori GPs to examine if the GPs’ personality and/or practicing style influence adherence to diabetes guidelines; c) see whether there is a cultural preference for a shared patient-doctor ethnicity; d) use data from GP clinic’s servicing different socioeconomic demographics.

Second, the small sample size (n=12) reflects the demographics of the family practice, and perhaps another family practice in a different suburb may yield a larger sample size than the potential (n=16). The exclusion of the other four participants was because of DAR's non-attendance two years before the study period, which in and of itself could exemplify disparities in healthcare uptake by Māori.

## Conclusions

Notwithstanding that a larger sample would have allowed stratification and helped reduce bias, study findings challenge the preconceived notion of economic disadvantage as a barrier to healthcare engagement while supporting difficulties in encouraging Māori to engage with the health system. Further, it excludes those without easy telephone coverage as some participants lived in remote areas with limited cell phone coverage if they did not have an existing landline (non-mobile house phone). A strength of the qualitative portion of the study is that it provided valuable cultural insights and a perspective into barriers to primary healthcare engagement. Trust remains a major influencer considering its consistent vocalizations in the interviews, outranking previous barriers such as cost, associated with low use of medical services or interventions.

## Appendices

Appendix 1

Consent template:

● Consent for participation in the study examining: Impact of covid on maintenance

follow-up of Māori patients with diabetes.

● I______________________ have had the reasons for the study clearly explained to me

and give consent to be interviewed or asked to fill a questionnaire or both. I consent

to Natalie Burkhardt having access to my medical records regarding my diabetes and

understand that this data will be used in the study. I understand that any data

collected is for the purpose of this study only, is strictly confidential and there will be

no information used that I can be identified by.

Signature: _______________________________Date: ___________________

Appendix 2

QUESTIONNAIRE

Please circle one of the options below which best rates the effect the statement has had on

attending appointments for your diabetes.

LACK OF TRUST IN HEATH SYSTEM 

Disagree Neutral Agree

PERSONAL FEAR OF CONTRACTING COVID-19:

Disagree Neutral Agree

FEAR OF CONTRACTING COVID AND INFECTING whānau:

Disagree Neutral Agree

FINANCE BARRIERS:

Disagree Neutral Agree

BECAUSE OF UNVACCINATED STATUS:

Disagree Neutral Agree

FACILITY NOT ACCESSIBLE OR UNABLE TO PROVIDE CONSULTS:

Disagree Neutral Agree

whānau COMMITMENTS TAKING PRIORITY OVER PERSONAL HEALTH:

Disagree Neutral Agree

PERCEIVED CHANGE IN DR-PATIENT RELATIONS DURING COVID:

Disagree Neutral Agree

NOT WILLING TO CONSULT ANOTHER GP IF USUAL GP IS UNAVAILABLE:

Disagree Neutral Agree
